# Staple Line Dehiscence in Gastric Conduit Following Esophagectomy: A Complication Conspicuously Missing a Mention It Deserves

**DOI:** 10.7759/cureus.21581

**Published:** 2022-01-25

**Authors:** Anvin Mathew, Gourav Kaushal, Deepti Ramachandra, Nirjhar Raj Rakesh, Puneet Dhar

**Affiliations:** 1 Surgical Gastroenterology, All India Institute of Medical Sciences, Rishikesh, IND; 2 Surgical Gastroenterology, All India Institute of Medical Sciences, Bathinda, IND

**Keywords:** conduit salvage, minimally invasive esophagectomy (mie), gastric conduit, staple line dehiscence, carcinoma esophagus, transthoracic esophagectomy

## Abstract

Following esophagectomy, anatomical reconstruction with a gastric tube is the most common practice. The construction of the gastric tube is done with staplers nowadays, be it a minimally invasive esophagectomy or a conventional open surgery. Even though anastomotic leak and conduit necrosis are reported widely in the literature, the number of studies on staple line dehiscence is meager in comparison. Management of conduit failure usually sacrifices conduit combined with a diverting cervical esophagostomy. We report a case of successful surgical management of a big staple line dehiscence and ‘salvaging of the conduit'.

## Introduction

The gastric pull-up is the most frequent method of reconstruction following esophagectomy. Either the whole stomach or a 2-6 cm wide gastric tube can be used [1]. Regardless of the technique, complications like anastomotic leak and conduit necrosis can occur. Literature on staple line dehiscence is conspicuously lacking, and the incidence rate is not known [2]. This may be due to clubbing of staple line dehiscence with anastomotic leak or conduit necrosis. A review of the available literature suggests managing staple line dehiscence either endoscopically or surgically, however, surgical management often ends up in conduit sacrifice and diversion. Here, we are reporting a case of successful surgical management of large staple line dehiscence without sacrificing the conduit.

## Case presentation

A 46-year-old gentleman presented with absolute dysphagia. On evaluation, diagnosis of poorly differentiated squamous cell carcinoma at 30 cm from incisors was established. He underwent laparoscopic feeding jejunostomy followed by neoadjuvant chemo-radiation with 41.5 Gy radiation and four cycles of paclitaxel and carboplatin. Twelve weeks following the completion of neoadjuvant therapy, the patient underwent an Mc-Keown esophagectomy. The thoracic part was video-assisted thoracoscopic surgery (VATS) assisted, and abdominal dissection and conduit construction were done via laparotomy. A 4 cm wide gastric tube was created using (3.8 mm) open linear cutting double-layer staplers; the staple line was not re-enforced with a suture. The esophagogastric anastomosis was performed in the neck.

**Figure 1:**
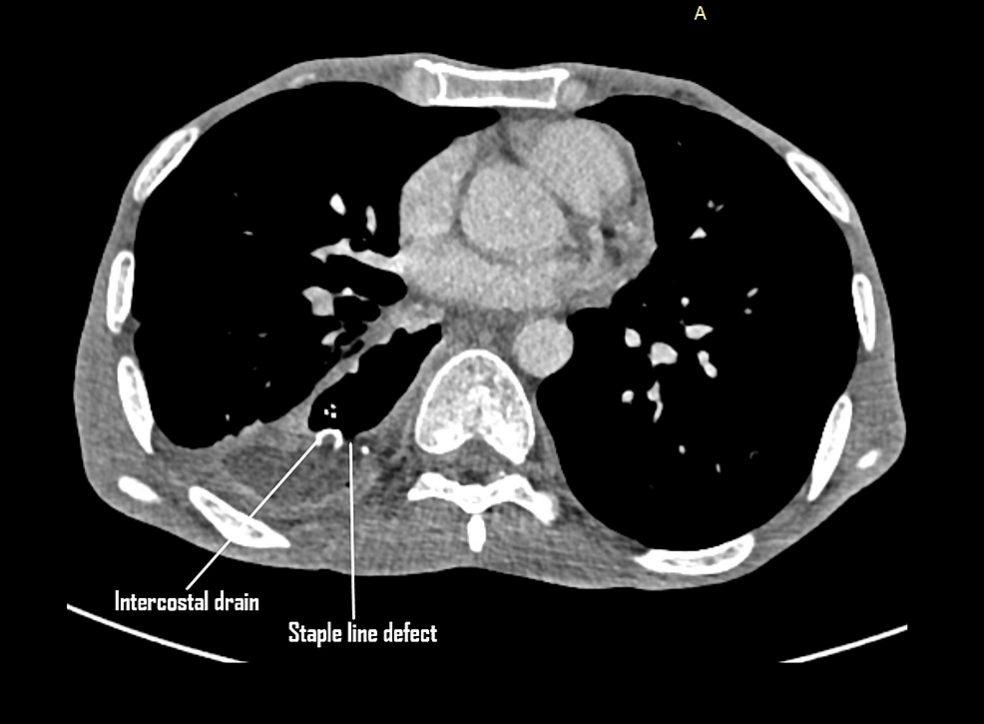
Axial cut from contrast-enhanced CT (CECT) thorax done on day nine showing an empyema in the right hemithorax. A defect in the conduit can be appreciated which is communicating with the empyema

The patient’s initial post-op recovery was smooth, and oral feeds were initiated by day five, and intraoperatively placed thoracostomy tube was removed on day six after surgery. However, he developed tachypnoea and fever spikes on day seven, associated with rising total leucocyte count (TLC). A chest X-ray suggested an effusion in the right hemithorax into which an ultrasound-guided percutaneous drain was placed. A contrast-enhanced CT (CECT) scan of the thorax on day 9 revealed a large empyema in the right thoracic cavity and a defect in the conduit which was freely communicating with the empyema (Figure 1).

**Figure 2:**
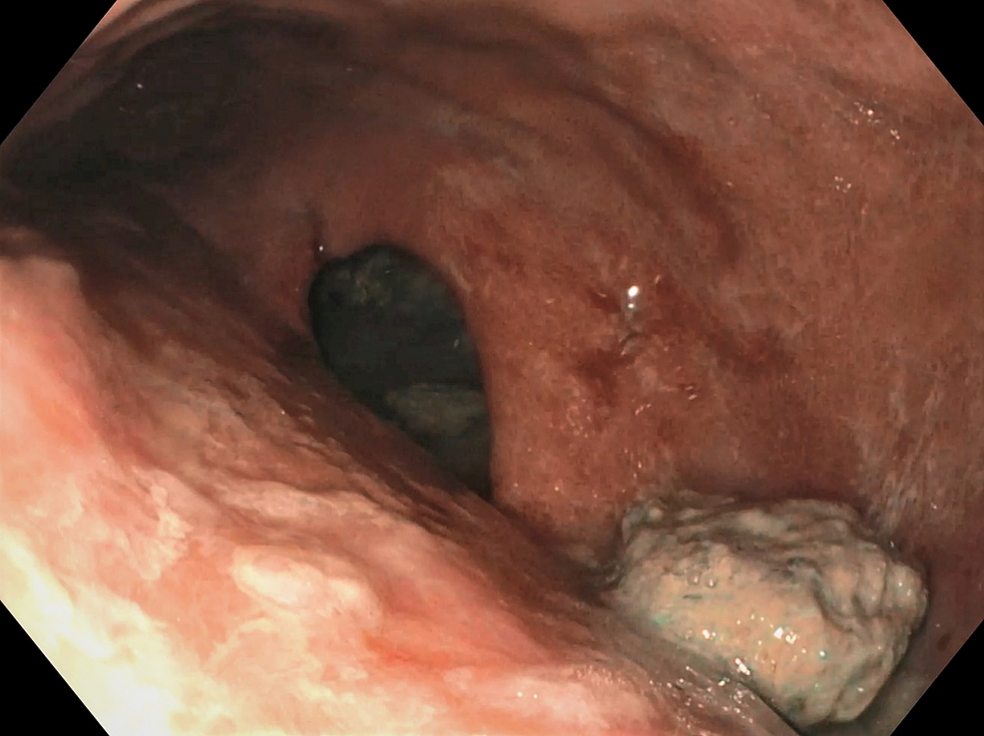
Endoscopic image showing an approximately 5 x 2.5 cm sized defect in the staple line

The septic episode was managed by replacing the percutaneous drain (PCD) with a 24F intercostal drain for better drainage and adding broad-spectrum antibiotics. Later an endoscopic assessment revealed an approximately 5 x 2.5 cm sized defect in the staple line (Figure 2).

**Figure 3:**
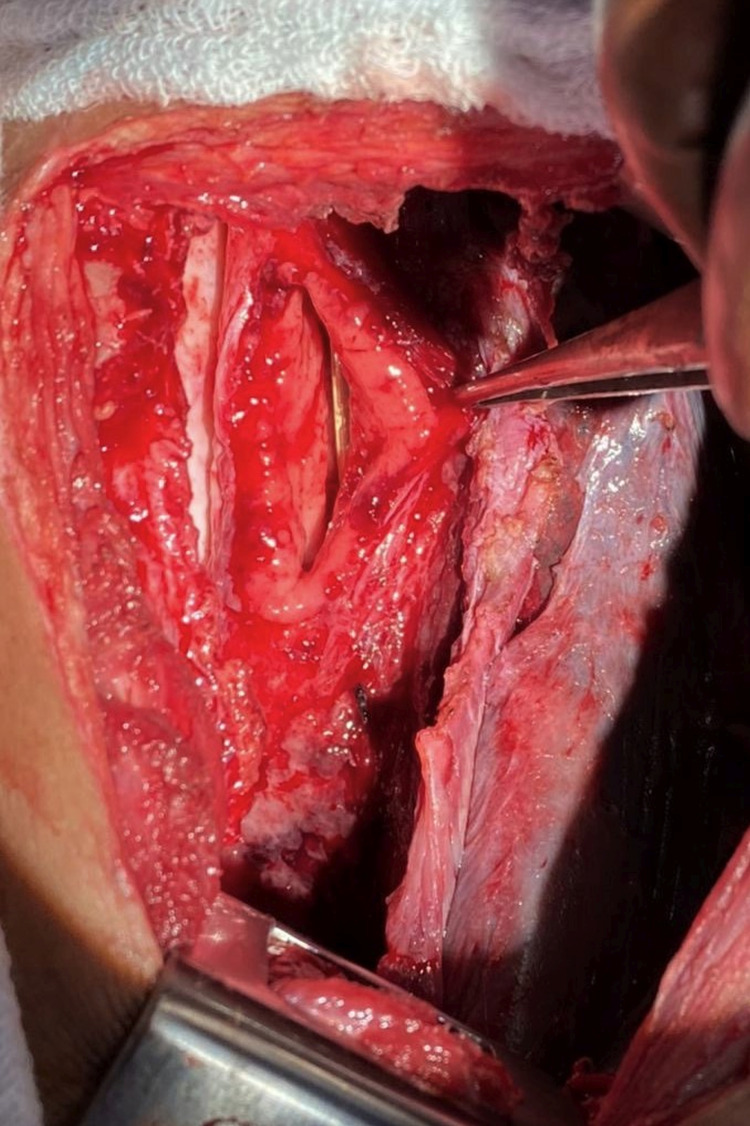
Intraoperative image showing a 5 x 2.5 cm sized defect along the staple line of the conduit

Various endoscopic options were considered but found unsuitable for this patient after discussion with the medical gastroenterologist. As the defect size was big, Over the scope clips (OTSC) clips could not be applied. Self-expanding metallic stent (SEMS) placement was also not undertaken as the biggest available diameter of SEMS available was 23 mm, while conduit diameter was 4 cm, and hence stent migration was inevitable. Due to a lack of viable endoscopic options, surgical repair of the defect was contemplated. The patient received aggressive nutritional and respiratory rehabilitation. He underwent the second surgery 46 days after the first; after the initial thoracoscopy, the right posterolateral thoracotomy approach was adopted due to dense adhesions and inflammation. Intraoperatively, a 5 x 2.5 cm sized defect was found along the staple line of the conduit (Figure 3).

**Figure 4:**
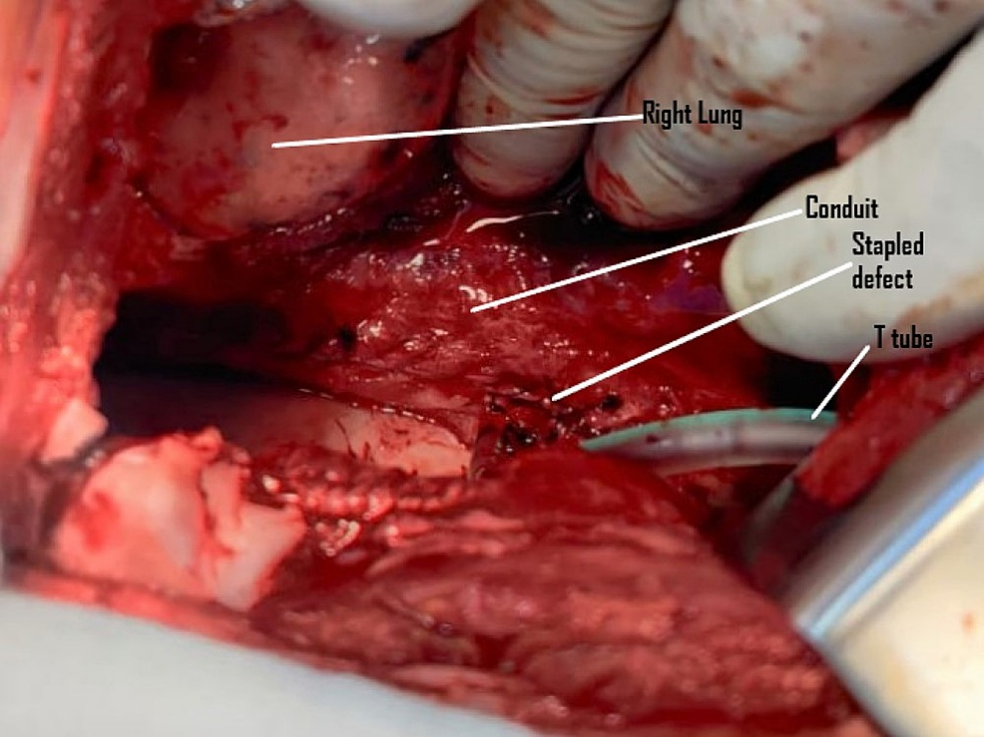
Post repair image. The stapled defect is marked. The T-tube which was inserted to decompress the conduit in the immediate post-operative period can also be seen

The defect was closed using Endo gastrointestinal anastomosis (GIA) staplers and was reinforced with polypropylene 4-0 suture. A 14 Fr T tube was placed through a separate opening to decompress the conduit in the initial post-op period to prevent a re-leak (Figure 4).

Thorough lavage of the cavity was performed with warm normal saline, and a 28 F tube was placed in the pleural cavity.

**Figure 5:**
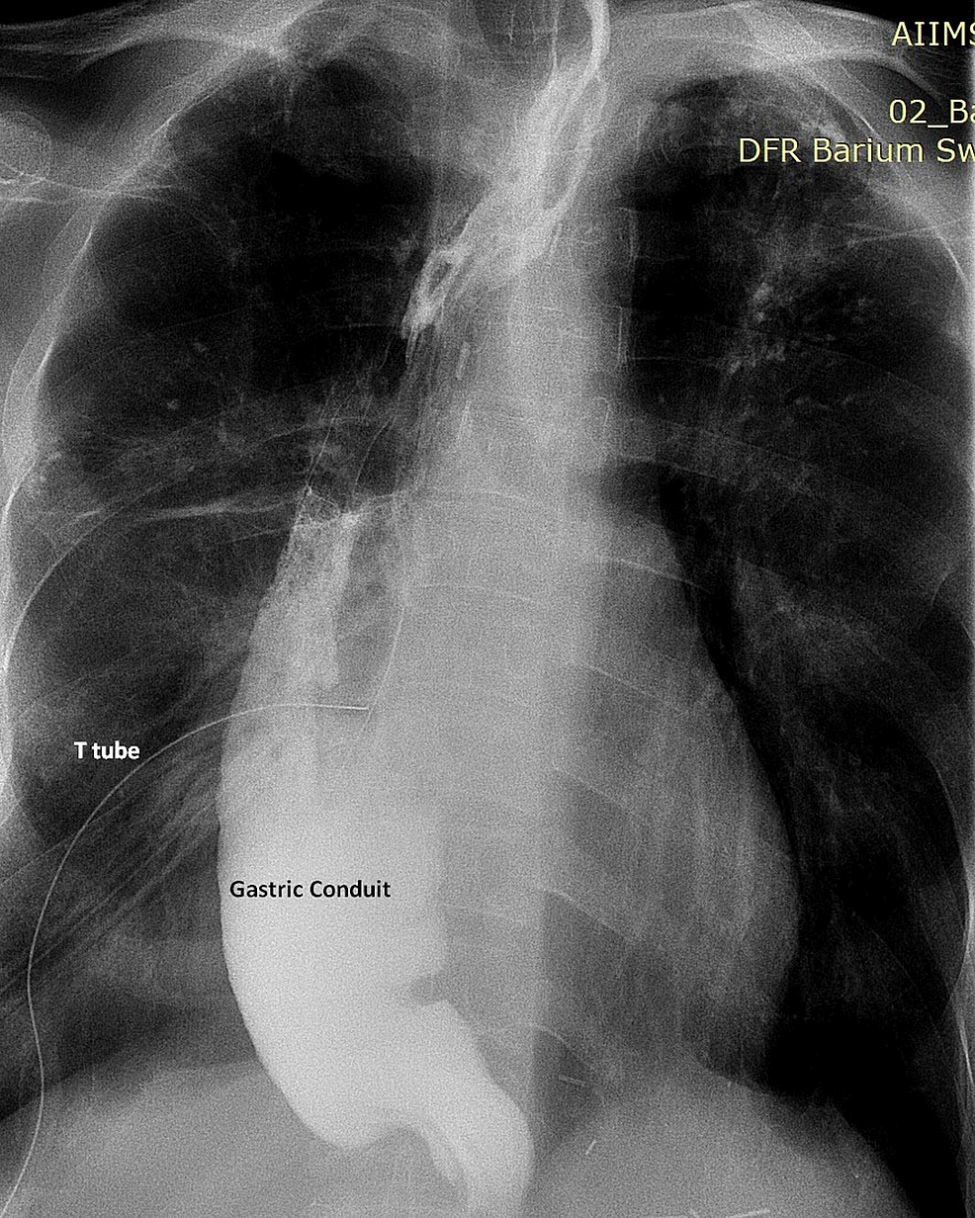
Post repair upper gastrointestinal contrast image showing no contrast leak

The patient had a prolonged seropurulent discharge from the drain. Bedside Methylene blue administered orally on day ten did not leak into the drain; the integrity of the repair was further verified by water-soluble upper gastrointestinal contrast series (Figure 5).

The patient was started on oral feeds and gradually progressed. The drain was irrigated with normal saline to facilitate the toileting of the pleural cavity. Gradually the drain output reduced, and the clinical condition of the patient improved. He was discharged with a chest tube drain in situ for home-based care, where the output from the drain eventually ceased. Unfortunately, the patient contracted COVID-19 infection at home and succumbed around three weeks after discharge.

## Discussion

Management of anastomotic leak and conduit necrosis are widely reported and discussed in the literature; however, the same is not valid for staple line dehiscence. The incidence rate of the staple line dehiscence is not clear [2]. Most of the available information is in the form of case reports. Silberhumer GR et al. reported a 2.7% incidence of staple line dehiscence in a cohort of 151 patients, and it is the only case series available in the literature. Out of the 151 patients studied, they had four staple line leaks, and all were seen in patients who did not undergo a staple line reinforcement. All four patients were managed surgically, but details of surgical interventions are not available [3]. We must remember that our patient also did not receive staple line reinforcement. Another report of two patients with staple line dehiscence was reported by Boone et al., in which the first patient was managed by drainage and thereby making a controlled fistula, whereas the second patient was managed surgically but had to succumb to the illness [2]. A few other case reports describe endoscopic SEMS placement as a modality of treatment [4,5]. However, considering the small diameter of available SEMS compared to the gastric tube diameter, the chances of stent migration would be very high in patients with wider gastric tubes.

The plausible explanation for the scarcity of data on staple line dehiscence is inaccurate clubbing of this entity with anastomotic leak and conduit necrosis. A recent meta-analysis on staple line dehiscence in sleeve gastrectomy calculated a staple line dehiscence rate of 1.5% [6]. So in gastric conduit also the incidence should be no less than that as both procedures involve a long staple line. Even though the recent Esophagectomy Complications Consensus Group (ECCG) listed out reporting strategies for various esophagectomy complications, staple line dehiscence is absent from the list [7]. In order to develop better management strategies for staple dehiscence, we need to have robust reporting systems which accurately capture the incidence of this condition.

## Conclusions

Staple line dehiscence is a life-threatening complication that is underreported/misreported in the literature. Management entails establishing a controlled fistula and control of sepsis followed by surgical repair/endoscopic methods. Reinforcement of staple lines with sutures may reduce the incidence of a leak. Developing a more robust reporting system and separating this entity from conduit failure/necrosis could aid in refining the treatment strategies.

## References

[1] Zhang W, Yu D, Peng J, Xu J, Wei Y (2017). Gastric-tube versus whole-stomach esophagectomy for esophageal cancer: A systematic review and meta-analysis. PLoS One.

[2] Boone J, Rinkes IH, van Hillegersberg R (2006). Gastric conduit staple line after esophagectomy: to oversew or not?. J Thorac Cardiovasc Surg.

[3] Silberhumer GR, Györi G, Burghuber C (2009). The value of protecting the longitudinal staple line with invaginating sutures during esophageal reconstruction by gastric tube pull-up. Dig Surg.

[4] Liang DH, Meisenbach LM, Kim MP, Chan EY, Khaitan PG (2017). Management of gastric conduit dehiscence with self-expanding metal stents: a case report on salvaging the gastric conduit. J Cardiothorac Surg.

[5] Oshikiri T, Yamamoto Y, Miki I (2015). Conservative reconstruction using stents as salvage therapy for disruption of esophago-gastric anastomosis. World J Gastroenterol.

[6] Gagner M, Kemmeter P (2020). Comparison of laparoscopic sleeve gastrectomy leak rates in five staple-line reinforcement options: a systematic review. Surg Endosc.

[7] Low DE, Alderson D, Cecconello I (2015). International consensus on standardization of data collection for complications associated with esophagectomy: Esophagectomy Complications Consensus Group (ECCG). Ann Surg.

